# Is there concordance between LV fibrosis and RV fibrosis in patients with hypertrophic cardiomyopathy; should there be?

**DOI:** 10.1186/1532-429X-16-S1-P313

**Published:** 2014-01-16

**Authors:** Nessim N Amin, Saundra Grant, June A Yamrozik, Ronald B Williams, Diane V Thompson, Moneal Shah, Mark Doyle, Robert W Biederman

**Affiliations:** 1 Cardiac MRI, Allegheny General Hospital, Pittsburgh, Pennsylvania, USA

## Background

CMR has become the leading modality to define the clinical impact of hypertrophic cardiomyopathy (HCM) providing complete coverage of both ventricles with high spatial resolution. Late gadolinium enhancement (LGE) accurately identifies regions of myocardial fibrosis. Via CMR, innumerable studies have established that LVH and LGE are the predominant phenotypic expressions of HCM. It is well known that myocardial fibrosis can occur in patients with HCM and is independently linked to a poorer prognosis than those without fibrosis by CMR.

## Methods

A retrospective review of all patients referred for HCM who underwent CMR was performed. SSFP/LGE techniques was used to diagnose patients with HCM, using gadolinium administration (0.15 mmol/kg, MultiHance, Bracco, Princeton-NJ), appropriate CMR phenotype, as well as clinical confirmation. Post-injection (10 minutes) LGE images were obtained using manual T1-weighted, IR-preparations. Regions of myocardium with LGE signals were visually designated as fibrotic. LV/RV mass indices (LVMI/RVMI) and ejection fractions were calculated. Fibrosis was semi-quantitatively assessed in both ventricles.

## Results

Via 72 patients referred for HCM from 2007-2013, 47(65%) were CMR and clinically confirmed to be HCM. The mean LVMI was 108 ± 44 g/m2 while the mean RVMI was 30 ± 21 g/m2. All patients met formal LVH criteria while 34/47 (72%) met RVH criteria. As well, 34/47(72%) had evidence of LV fibrosis while 24/47 (51%) had evidence for RV fibrosis. Only 2 patients with RV fibrosis had absent LV fibrosis. Of the RVH positive patients, 26/34(76%) patients were LV LGE positive and 18/34 (52%) were RV LGE positive.

## Conclusions

The high frequency of RVH and RV fibrosis in the setting of HCM is surprising in that this phenomenon is rarely described. However, given the genetic abnormalities, there is no reason to expect the phenotypic expression should be limited to the LV. Interestingly, as for the LV, the presence or absence of RV fibrosis had little predictive power towards the systolic function. HCM effects *both *the LV and the RV.

## Funding

Internal.

